# Increasing the efficiency of bacterial transcription simulations: When to exclude the genome without loss of accuracy

**DOI:** 10.1186/1471-2105-9-373

**Published:** 2008-09-12

**Authors:** Marco AJ Iafolla, Guang Qiang Dong, David R McMillen

**Affiliations:** 1 Department of Chemical and Physical Sciences and Institute for Optical Sciences, University of Toronto Mississauga, 3359 Mississauga Rd N, Mississauga, ON, L5L 1C6, Canada

## Abstract

**Background:**

Simulating the major molecular events inside an *Escherichia coli *cell can lead to a very large number of reactions that compose its overall behaviour. Not only should the model be accurate, but it is imperative for the experimenter to create an efficient model to obtain the results in a timely fashion. Here, we show that for many parameter regimes, the effect of the host cell genome on the transcription of a gene from a plasmid-borne promoter is negligible, allowing one to simulate the system more efficiently by removing the computational load associated with representing the presence of the rest of the genome. The key parameter is the on-rate of RNAP binding to the promoter (k_on), and we compare the total number of transcripts produced from a plasmid vector generated as a function of this rate constant, for two versions of our gene expression model, one incorporating the host cell genome and one excluding it. By sweeping parameters, we identify the k_on range for which the difference between the genome and no-genome models drops below 5%, over a wide range of doubling times, mRNA degradation rates, plasmid copy numbers, and gene lengths.

**Results:**

We assess the effect of the simulating the presence of the genome over a four-dimensional parameter space, considering: 24 min <= bacterial doubling time <= 100 min; 10 <= plasmid copy number <= 1000; 2 min <= mRNA half-life <= 14 min; and 10 bp <= gene length <= 10000 bp. A simple MATLAB user interface generates an interpolated k_on threshold for any point in this range; this rate can be compared to the ones used in other transcription studies to assess the need for including the genome.

**Conclusion:**

Exclusion of the genome is shown to yield less than 5% difference in transcript numbers over wide ranges of values, and computational speed is improved by two to 24 times by excluding explicit representation of the genome.

## Background

In recent decades, extraordinary advances in biochemistry and molecular biology have led to an unprecedented level of understanding biological systems at the molecular level. The complexity of cellular pathways and networks often makes it difficult or impossible to reliably predict the behavior of a system from knowledge of its components, and thus there is considerable interest in formulation of quantitative, predictive mathematical models of cellular functions. Such efforts, collectively described by such terms as systems biology and *in silico *biology [1-9], aim in the long term toward goals such as predicting the effects of drugs or other interventions on the state of diseased cells, and enhancing our fundamental understanding of how cells respond to stimuli and regulate their internal environments.

The internal dynamics of cells are driven by the kinetics of a complex set of biochemical reactions: the state of the cell may be viewed as the numbers and binding states of all species of interest, and the time evolution of that state is defined by how those species react with one another. A central challenge in cellular modelling is to formulate correct biochemical reaction schemes to represent a process of interest, and then to populate the reaction system with appropriate rate constants [5-9]. Within this effort, two persistent difficulties arise: populating mathematical models based on incomplete experimental information [10,11]; and the computational demands of simulating the resulting systems, which can grow large for even moderately complex processes.

We have previously carried out a study aimed at the first of these problems, in which we used bulk expression data from *Escherichia coli *to deduce the numbers of free RNA polymerases available to transcribe a target gene of interest [10]; this information is not currently experimentally available, with bulk studies [12] able to provide the average numbers of each enzyme type but not to determine how many are "tied up" in the cell, transcribing other genes, at any given time. When simulating the expression of a gene or network of genes, whether an engineered or "synthetic" system [13-18], or a natural one [2,6,8,19-21], the total number of RNA polymerases is less relevant than the number that are not currently occupied expressing genes outside the target system of interest. Our method for deducing this number involved using bulk measurements (collected as a function of growth rate [12]) to create an average (or "mean field") behaviour for the set of genes in the bacterial genome; we then tested how many expression enzymes our target gene had available to transcribe it, and generate free enzyme levels as a function of growth rate [10].

We turn now to the second of the challenges mentioned above, that of computational time. Having the rest of the genome present in the system, even in our bulk-averaged way, added significantly to the computational demands of the simulations. Further investigation shows, however, that there are regimes in which the target system is not significantly affected by the presence of the remainder of the genome, and may thus well approximated by excluding the genome portion and simulating only the target system. The key quantity is the "on rate" of binding between RNA polymerase and the promoter of the target gene: for certain ranges of this parameter, the perturbation introduced by the presence of other genes (the rest of the genome in the cell) is small enough to be neglected, saving significant amounts of computational time. We explore the details of these ranges, as a function of other system parameters, below. We view this work as complementary to the various ongoing large-scale cellular simulation projects [2,7,19,22-25], offering a method of simplifying the system in cases where including genes outside the immediate system of interest does not alter the overall behaviour significantly. Although our results are obtained for our particular gene expression model, we anticipate that our promoter on-rates will apply, at least approximately, to other studies of transcription in bacteria, and thus offer guidance to others wishing to simplify their system by omitting the genomic influence.

## Methods

### *E. coli *gene expression model

Our technique relies on the existence of experimental results [12] reporting bulk average assays of the amounts of each species present in the biological system of interest, as a function of growth rate; quantities such as average RNA polymerase per cell, average transcript content per cell, and so on, are much more readily obtained than specific rate constants for individual reactions. Using the bacterium *Escherichia coli *as a model organism, we have formulated a picture of the biochemical reactions underlying gene expression from an inserted plasmid carrying a promoter controlling the transcription of our target gene. We implemented a "mean-field" modelling approach, generating genome-wide averages for the mean transcript length, mean elongation time, and so on, adjusting the model parameters so that it generated numbers matching the bulk averages that had previously been reported experimentally [10,12]. A full list of the reactions included in the model and the nomenclature used for the species is provided in Tables 1, 2, and 3. The following sections provide an overview of the processes represented in the model, with further details provided in the Appendix and in our previously published work [10].

**Table 1:** Biochemical reactions that make up our bacterial gene expression model (version incorporating the host's genome).

Left	Right	Forward Rate constant	Backward rate constant
operon_ns + Rpoly	closed_Rpoly_prom_ns	k_on_Rpoly_prom_ns/(v/1000)	k_off_Rpoly
closed_Rpoly_prom_ns	open_Rpoly_prom_ns	k_isomerization	
open_Rpoly_prom_ns	operon_ns + Rpoly_operon_ns_1	k_prom_clearance	
Rpoly_operon_ns_1	Rpoly_operon_ns_2 + mRNA	k_transcription_ns	
Rpoly_operon_ns_2	Rpoly_operon_ns_3 + mRNA	k_transcription_ns	
Rpoly_operon_ns_3	Rpoly_operon_ns_4 + mRNA	k_transcription_ns	
Rpoly_operon_ns_4	Rpoly_operon_ns_5 + mRNA	k_transcription_ns	
Rpoly_operon_ns_5	Rpoly_operon_ns_6 + mRNA	k_transcription_ns	
Rpoly_operon_ns_6	Rpoly_operon_ns_7 + mRNA	k_transcription_ns	
Rpoly_operon_ns_7	Rpoly + mRNA_small	k_transcription_ns/0. 871794871794871	
operon_s + Rpoly	closed_Rpoly_prom_s	k_on_Rpoly_prom_s/(v/1000)	k_off_Rpoly
closed_Rpoly_prom_s	open_Rpoly_prom_s	k_isomerization	
open_Rpoly_prom_s	operon_s + Rpoly_operon_s	k_prom_clearance	
Rpoly_operon_s	Rpoly + stable_RNA	k_transcription_s	
V	2v	k_cell_div	
	operon_ns	k_rep_operon_ns	
	operon_s	k_rep_operon_s	
	Rpoly	k_rep_Rpoly	
plas + Rpoly	closed_Rpoly_prom_reporter	k_on_Rpoly_prom_reporter/(v/1000)	k_off_Rpoly
closed_Rpoly_prom_reporter	open_Rpoly_prom_reporter	k_isomerization	
open_Rpoly_prom_reporter	plas + Rpoly_reporter + incom_mRNA_reporter	k_prom_clearance	
Rpoly_reporter	Rpoly + mRNA_reporter	k_transcription_reporter	
incom_mRNA_reporter		k_transcription_reporter	
mRNA_reporter		k_deg_mRNA_reporter	
incom_mRNA_reporter		k_deg_mRNA_reporter	
Rpoly_reporter	Rpoly	incom_mRNA_reporter*k_deg_mRNA_reporter/Rpoly_reporter	

Gene expression model incorporating the host's genome. Lists of the biochemical reactions that make up our bacterial gene expression model, for the version of the model that includes an "averaged" version of the host cell's genome. Columns Left and Right represent the left and right sides of chemical reactions, and the Forward and Backward rate constants are associated with the forward and reverse reactions.

**Table 2:** List of the biochemical reactions that make up our bacterial gene expression model (version excluding the host's genome). Gene expression model excluding the host's genome

Left	Right	Forward rate constant	Backward rate constant
V	2v	k_cell_div	
	Rpoly	k_rep_Rpoly	
plas + Rpoly	closed_Rpoly_prom_reporter	k_on_Rpoly_prom_reporter/(v/1000)	k_off_Rpoly
closed_Rpoly_prom_reporter	open_Rpoly_prom_reporter	k_isomerization	
open_Rpoly_prom_reporter	plas + Rpoly_reporter + incom_mRNA_reporter	k_prom_clearance	
Rpoly_reporter	Rpoly + mRNA_reporter	k_transcription_reporter	
incom_mRNA_reporter		k_transcription_reporter	
mRNA_reporter		k_deg_mRNA_reporter	
incom_mRNA_reporter		k_deg_mRNA_reporter	
Rpoly_reporter	Rpoly	incom_mRNA_reporter*k_deg_mRNA_reporter/Rpoly_reporter	

Lists of the biochemical reactions that make up our bacterial gene expression model, for the version of the model that excludes the host cell's genome. Columns Left and Right represent the left and right sides of chemical reactions, and the Forward and Backward rate constants are associated with the forward and reverse reactions.

**Table 3:** Species nomenclature used in biochemical models

Species Label	Species
Rpoly	RNA polymerase
closed_Rpoly_prom_ns*	RNA polymerase in a closed-complex with the average genomic mRNA operon promoter
closed_Rpoly_prom_s*	RNA polymerase in a closed-complex with the average genomic sRNA operon promoter
closed_Rpoly_prom_reporter	RNA polymerase in a closed-complex with the reporter mRNA promoter on the plasmid
open_Rpoly_prom_ns*	RNA polymerase in an open-complex with the average genomic mRNA operon promoter
open_Rpoly_prom_s*	RNA polymerase in an open-complex with the average genomic sRNA operon promoter
open_Rpoly_prom_reporter	RNA polymerase in an open-complex with the reporter mRNA promoter on the plasmid
Rpoly_operon_ns*	RNA polymerase elongating the average genomic mRNA transcript from the average genomic mRNA operon
Rpoly_operon_s*	RNA polymerase elongating the average genomic sRNA transcript from the average genomic sRNA operon
Rpoly_reporter	RNA polymerase elongating the reporter mRNA transcript from the plasmid
incom_mRNA*	Nascent average genomic mRNA
incom_mRNA_small*	Nascent genomic mRNA where its final length is approximately 90% of the average genomic mRNA
incom_mRNA_reporter	Nascent reporter mRNA
mRNA*	Average genomic mRNA (represented as the length of the average genomic mRNA gene)
mRNA_small*	Genomic mRNA that is approximately 90% of the average genomic mRNA
stable_RNA*	Average genomic sRNA (represented as the length of the average genomic sRNA operon)
mRNA_reporter	Reporter mRNA – the mRNA of interest
operon_ns*	Average genomic mRNA operon
operon_s*	Average genomic sRNA operon
plas	Reporter promoter on the plasmid
v	Cell volume
*	Refers to species used exclusively in the gene expression model that incorporates the host's genome

List of species names used in the two versions of the model.

#### Cell growth and division

The cellular volume grows exponentially until a threshold is reached, at which point it is approximately halved (a binomial distribution is used) and exponential growth restarts. A counter species, *v*, is used to represent volume: *v *→ 2*v*, with rate constant *k *= ln(2)/τ, where τ is the doubling time of the cells. At cell division, all species present are divided between two hypothetical daughter cells, and the simulation follows one of these daughters. We treat the system as ergodic, and average over long times for a single cell to obtain ensemble averages across the cellular population.

#### Enzyme binding and isomerization

RNA polymerases (Rpoly) are responsible for initiating and catalyzing the transcription of messenger RNA (mRNA) strands. As the model assumes all mRNA transcripts reside in operons, Rpoly binds to promoter sequences in the DNA (operon) and forms a closed complex (Rpoly+operon→closed_Rpoly_prom). This closed complex then must isomerize into an open complex (closed_Rpoly_prom→open_Rpoly_prom) before transcription can begin.

#### Enzyme clearance

RNA polymerases clear the promoters, leaving those sites free to bind additional enzymes while transcription proceeds further down the DNA strand. We model this by regenerating the promoter after clearance occurs, forming an enzyme-template complex plus the original site: open_Rpoly_prom→Rpoly_operon+incom_mRNA+operon. We create a nascent transcript (mRNA_incom) at this step to allow subsequent translation to proceed; this feature will prove very helpful in studying future simulated studies of protein synthesis. Conservation of the number of promoters is maintained: when the enzyme-template complex finishes elongation, only the enzyme and the polymerized product are released.

#### Elongation

To avoid the complexity of accounting for each enzyme at different stages of elongation, a single reaction is used to represent the process of completing the mRNA chain: Rpoly_operon→Rpoly+mRNA. Compliment to this reaction is the disappearance of the nascent transcript made available during transcription: incom_mRNA→(), where () is a null placeholder. Both the reactions have the same elongation rate constant that can be summarized as k_elongation _= ρ/λ, where ρ and λ are the polymerization rate and length of template, respectively.

#### Enzyme production

Since the kinetics of RNA polymerase assembly are not fully known, the model is simplified by treating enzyme production as a zero-order process in which enzymes appear from outside the model at a constant rate: ()→Rpoly. The enzymes are partitioned at cell division like all other species. The rate constant for production can be summarized as k_rep _= (ν/1.5)/τ, where ν and τ are the average number per cell and cellular doubling time, respectively.

#### DNA replication

DNA replication in bacteria is a complex process involving multiple replication forks. We represent the coding portion of the genomic DNA by the number of operons present (operon), and simplify the replication process as a zero-order process: ()→operon. Rate constants for this process are chosen to match the number of genomes per cell at different growth rates.

#### mRNA degradation

RNases act to destroy mRNA in *E. coli*, and we represent the degradation of mRNA by these enzymes with first-order reactions: mRNA→(), and incom_mRNA→(); the latter is an additional RNase-driven degradation, beyond the above-mentioned rate of disappearance of incomplete mRNA through conversion to complete mRNA strands.

#### RNA production from operons

We assume that all genes in the genome are clustered into operons: groups of genes transcribed from a single promoter, as in the *lac *operon. The model keeps track of which gene on the mRNA operon Rpoly is currently transcribing and makes available completed transcripts of the nascent operon (this latter point will prove relevant in future protein synthesis models): Rpoly_operon1→Rpoly_operon2+mRNA. In response to the genome-wide average of 6.9 genes per operon [10,12] the model tracks the 7 transcripts representing the average mRNA operon (six genes of equal size, one 90% the length of the average size).

In addition to messenger RNA, other forms of RNA collectively known as stable RNA (sRNA) are produced within the cell. Since sRNA is transcribed but not translated the model does not consider nascent sRNA production.

##### With-genome and no-genome models

**Figure 1:**
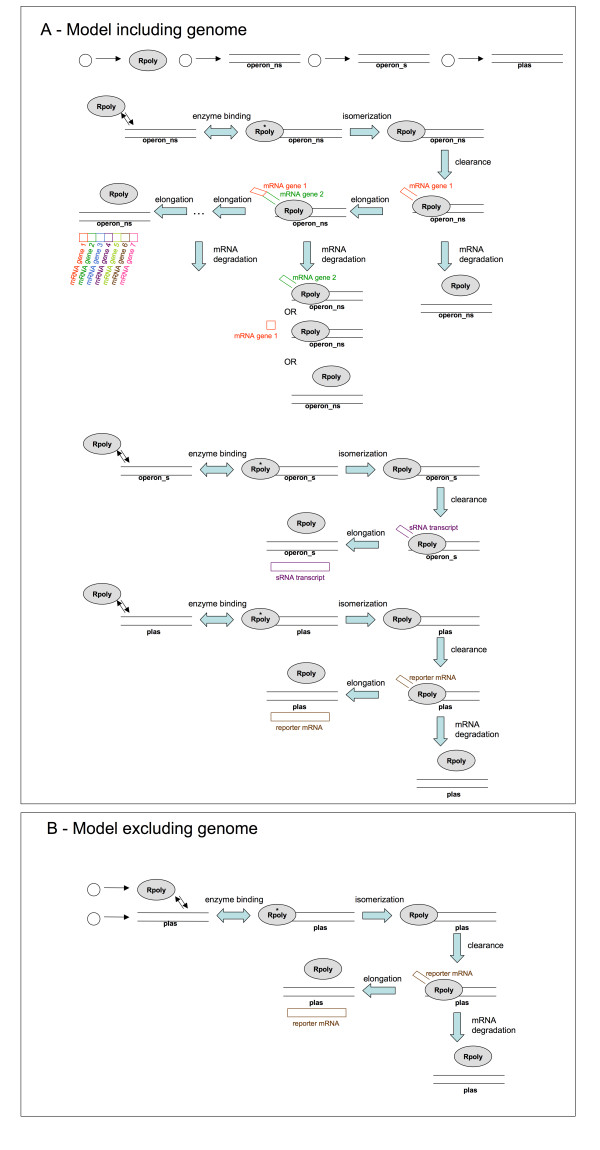
**Schematic of the two versions of the model system**. Our simulations compare two versions of a bacterial gene expression model. (A) In the first version, the genome is represented as an "average" open generating generic transcripts, rather than as the full set of individual genes. Bulk experimental measurements are used to generate the correct average number of transcripts from this generic operon in the genome. In this version of the model, the promoter residing on a plasmid of interest (plas) competes with the genomic operons for access to RNA polymerase (Rpoly) enzymes. (B) In this version, all references to the host cell genome are excluded from the model, leaving only the plasmid-borne promoter (plas) to be transcribed by RNA polymerase (Rpoly). Full lists of the reactions that constitute the models are given in Tables 1 and 2, with a list of species names given in Table 3.

We have constructed two versions of the model, one containing a representation of the host cell genome and the reporter gene, the other neglecting the cellular genome and representing only the reporter gene on the plasmid. The with-genome model incorporates 26 reactions involving 27 species, while the no-genome version has 10 reactions involving 10 species; the two versions are shown schematically in Figures 1A and 1B. The genome affects a plasmid-borne gene of interest by competing for RNA polymerase binding with the plasmid-borne promoter, while in the no-genome version of the model we omit the genomic promoter sites and thus this competition does not occur. The goal, then, is to determine the parameter regimes in which this omission has an acceptably small influence on the behaviour of the system, and to determine how much more quickly the computational simulations will run as a result of the simplification.

##### Computational simulation method

The chemical kinetics of this system were initially simulated using the Gillespie Monte Carlo algorithm [26-28], and these results were used to validate a deterministic, ordinary differential equation (ODE) version of the system, which was shown to yield identical average transcript numbers, allowing us to use the significantly faster ODE model to generate larger numbers of points in parameter space. Comparing the two models allowed us to determine the point at which the on-rate constant between the target promoter and RNA polymerase, k_on, crossed a threshold where the two models (with and without the host genome included) generated average transcript numbers differing by more than a certain percentage; here, we have chosen a five percent difference as an admittedly arbitrary significance threshold.

The original experimental measurements in the literature were carried out over a range of cellular growth rates, each of which yielded different average quantities of biomolecules per cell. Stochastic simulations of our system were carried out at each experimentally-examined growth rate (doubling times of 24, 30, 40, 60, and 100 minutes [12]) and sampled at discrete points in parameter space, as follows: plasmid copy numbers of 10, 100, and 1000; mRNA half-lives of 2, 6, 10, and 14 min; and gene lengths of 10, 100, 1000, and 10000 bp. The relationship between these independent variables and the point at which the promoter-RNAP on-rate begins to yield a significant difference between the genome and no-genome models is complex and highly nonlinear, and not amenable to reduction to a single equation. We have instead produced a MATLAB script (The MathWorks, Natick, MA) that generates an on-rate threshold given a user's input of plasmid copy number, mRNA degradation rate, gene length and cellular doubling time: any promoter on-rate constant larger than this predicted value can exclude the computationally expensive genome from the simulations without creating more than a five-percent error, while any constant smaller than this should include the genome.

### Stochastic modelling approach and software

Deterministic chemical kinetics apply in the regime of large numbers of randomly interacting molecules. Inside cells, molecule numbers are often small enough to produce significant fluctuations [8,20,28-44], thus requiring a stochastic simulation of the reaction kinetics. The Gillespie algorithm [2] treats chemical reactions as Poisson processes, with event (reaction) rates given by microscopic rate constants and the current state of the system. For an elementary reaction of the form A+B→C with rate constant *k*, the Poisson rate of the forward reaction is *kab*/*V*, where *a *and *b *represent the numbers of molecules of species A and B present, and *V *is the reaction volume (note that this volume is a changing parameter in a living bacterial cell). We use the unit "n" to represent the number of molecules present in the system, rather than concentration units such as molarity. To advance the simulation, the timing of the next reaction event is randomly selected using the exponential distribution of inter-event times for the set of Poisson processes representing the reactions, and the probability of each reaction being the one that occurs at that instant is given by its fraction of the sum of all reaction rates [26-28].

Bacterial cells have often been approximated as well-stirred reactors: based on their small size, it is assumed that diffusion is sufficiently fast to yield a well-mixed system. Early experimental results showed protein mobility *in vivo *consistent with normal diffusion [45], and though the diffusion coefficients were substantially lower than for the same proteins in water, the diffusion was fast enough to spread the proteins over the volume of a bacterium on a time scale of seconds. Recent theoretical treatments [43,46-49] have questioned the picture of bacterial cells as well-mixed systems, and recent experimental results [50] have reported subdiffusive behavior in the motion of individual RNA molecules, where each RNA is rendered visible through binding to multiple fluorescent protein labels. In this paper, we use the well-stirred reactor picture as a first approximation to gain insight, but it should be noted that this is a significant simplification, and that future refinements and extensions are possible. Approaches proposed to deal with crowded cellular environments include rate laws obeying fractal-like kinetics [49,51,52], and Monte Carlo simulations wherein two- or three-dimensional spatial information is retained for each molecule [43,46,49,53].

The gene expression model was initially implemented using BioNetS (Biochemical Network Stochastic Simulator) [26], which provides a convenient interface for specifying reactants, products and kinetic data. The software generates C++ source code implementing the system using the Gillespie stochastic simulation algorithm (or an approximation, if desired), and this code is then compiled and executed with user-tunable parameters as inputs. Some species in the model exist in small numbers while others exist in large numbers; although continuum approximations and hybrid schemes are available through BioNetS [26], the Gillespie algorithm with no approximations yielded the best simulation speed. The data from the BioNetS-generated code was processed using DataTask (Visual Data Tools, Inc) and its run manager DataTask, which automated the process of sweeping parameter values and analyzing the results. The complete gene expression models used are available as BioNetS scripts and are provided along with this paper (see Additional File 1).

### Derivation of *E. coli *gene expression parameters

To derive the on-rate constant between RNA polymerase and the reporter promoter where there is 5% difference in transcript average between models, we employ bulk cellular averages obtained by Bremer and Dennis for several different cellular growth rates [12]. We implement a "mean-field" approach [10] by considering the production of generic transcripts with properties derived from genome-wide averages: we compute mean transcript lengths, mean elongation rates, and so on. With these quantities in hand, the unknown between models is reduced to the RNA polymerase on-rate constant for binding to the reporter promoter, and we find its value by sweeping until the difference in transcript average between models is 5%.

The model has been constructed to be as detailed as possible, using all available information about the biochemical processes underlying gene expression. This leads to a large number of species and reactions, the full details of which are provided in the Appendix. For a derivation of average genome parameters, please see Iafolla and McMillen [10].

### Stochastic model parameter sweeping

**Figure 2:**
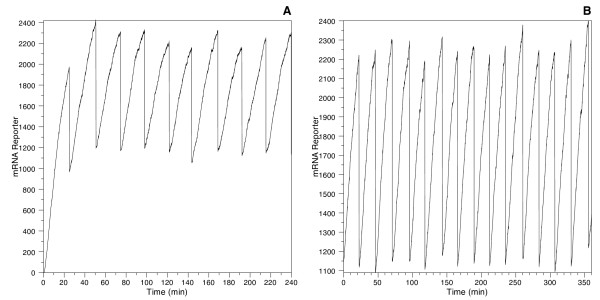
**Typical time series generated by the model**. Typical time series generated by the model. Plot A (left) shows a run with all intermediates and products initially set to zero, illustrating the initial transient. Plot B (right) shows a run initialized with the state obtained after 10 cell divisions in the left-hand run, thus removing the initial transient. Simulations were performed at a variety of cellular growth rates with different kinetic parameters. Parameters for this example: doubling time = 24 min; on-rate constant = 10^-3 ^n^-1^s^-1^; plasmid copy number = 10; gene length = 10^4 ^bp; and mRNA half-life = 14 min.

The first step in deriving the on-rate constant that determines a 5% difference in transcript averages between models is to obtain steady-state values of all species in the simulations. Figure 2 shows the time series for one species in the model, the reporter mRNA. An initial run of 10 cell divisions in length is generated for each simulation, and the final state of this run is used as the initial state for the long-duration run in which statistics are accumulated to determine average species levels; this prevents the initial transient approach to steady state from distorting the averages.

**Figure 3:**
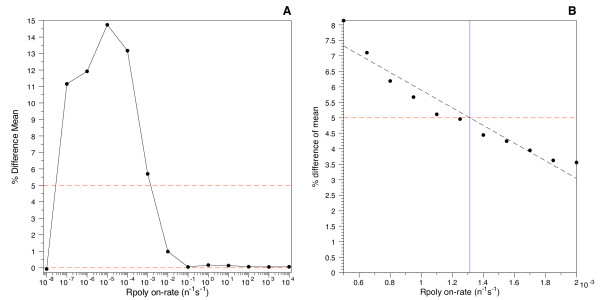
**Parameter sweeping**. Parameter sweeping. Here, we compare two versions of the gene expression model, one incorporating the host cell genome and one excluding it. The RNA polymerase on-rate constant for binding to the promoter that produces the reporter mRNA is varied until the percentage difference between these models exceeds 5% (the value we have selected as our threshold for a significant difference between the two models, marked by a horizontal dashed line on each plot). The on-rate is first varied by a factor of 10 to determine the general location of the desired value (plot A, left), followed by a sweep on a finer scale to narrow in on an approximately linear region near the threshold crossing (plot B, right). The solid vertical line in Graph B shows the interpolated on-rate constant when the percent difference in transcript production between models crosses the 5% threshold. The parameters for this example are: doubling time = 24 min; plasmid copy number = 10; gene length = 10 bp; and mRNA half-life = 6 min.

Parameter sweeping begins by using on-rates that vary by a factor of 10 (Figure 3A). When the desired percent difference between models lies between two on-rate constants, another sweep is performed between these new limits incrementing the on-rate by a unit multiple of the smaller limit. The third parameter sweep uses a unit increment of the next significant digit between the new limits; this change in on-rate is small enough to approximate linearity (Figure 3B). Only *R*^2 ^≥ 0.90 were accepted for interpolation; the range was narrowed until this level of linearity was achieved.

The duration of the stochastic simulations was varied to obtain linearity with R^2 ^≥ 0.90; this is achieved by using a minimum of 1000 cell divisions, although some simulations use more cell divisions to obtain the desired linearity. Since the doubling time of the cells is varied, the total duration in real time varies among the simulations; the number of cell divisions explored appears to be the key factor in obtaining well-converged statistics, rather than the absolute duration.

The minimum 1000 cell division duration was deduced by qualitative analysis of multiple simulations with the same seed but different durations; we examined the effect of duration on the mean values obtained from the reporter mRNA histograms. The on-rate constants used in the duration analysis was determined by comparing the histograms between models over a range of on-rate constants (10^-7 ^n^-1^s^-1 ^to 1 n^-1^s^-1^); the range of on-rate constants that bound the percent difference in the above statistical parameters by 5% was investigated for duration analysis (this range was from 10^-5 ^n^-1^s^-1 ^to 10^-2 ^n^-1^s^-1^). Ultimately, longer-duration runs produced averages that were not statistically different from those obtained after 1000 divisions (see the Appendix for additional explanation), implying that longer durations only increase computational expense.

After interpolation, the validity of the on-rate was tested: using a different seed for 30 simulations – all employing steady-state initial conditions and the same duration, kinetics and interpolated on-rate – the sample mean difference between models of the 30 simulations was statistically compared to the population mean of 5%. The on-rate was accepted if the two means were not proven statistically different using a level of significance α = 0.95. All simulations, either in parameter sweeping or verification, employ different nucleating random number generator seeds.

### Additional deterministic simulations

**Figure 4:**
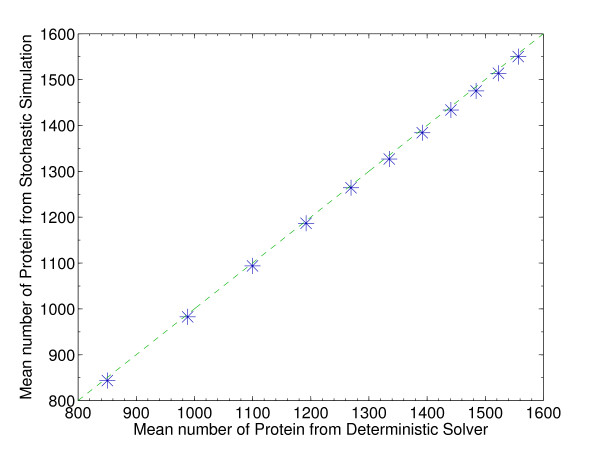
**Comparison of stochastic and deterministic simulation outputs**. Comparison of stochastic and deterministic simulation outputs. The stochastic simulations required too much computational time for it to be practical to sample the parameter space very densely. Since we have used only the mean values from the stochastic simulations, we explored the possibility of using deterministic simulations, which require a tiny fraction of the stochastic simulation time, to increase our sampling of the parameter space. The plot shows the average number of mRNA transcripts generated by the two methods, stochastic and deterministic. The straight diagonal line indicates a good match, and in fact the two methods differ by less than one percent in most cases. Parameter values are the same as those used in Figure 2A.

The stochastic simulations are very computationally intensive, and thus we investigated methods of speeding up the calculations. The ordinary differential equations corresponding to the full reaction system for each model (genome and no genome) were derived using standard chemical kinetics and solved numerically using the solvers provided by MATLAB. To take cell growth and division into account, the ODEs were solved one cell cycle at a time, with the numbers of molecules at the end of the cycle cut in half to simulate division, then used as the initial state for the next cell cycle. Within each set of parameter values, each ODE was run for ten cell cycles to allow the system to reach a steady state, then for more ten more cell cycles, during which state values were averaged to obtain the average mRNA numbers for the reporter gene. As shown in Figure 4, the average mRNA numbers from the stochastic simulations matched nearly perfectly with those generated by the ODEs, and on this basis we used the deterministic ODEs to increase the number of points in the parameter space that could be feasibly sampled. (This reduction to the deterministic model is possible because here we are considering only the mean values from the stochastic simulation; in cases where the fluctuations were the point of interest, fully stochastic simulations would of course be required.) Full-scale stochastic simulations were carried out for the experimentally available doubling times (24, 30, 40, 60, and 100 minutes [12]), varying the other parameters as follows: gene lengths of 10, 100, 1000, and 10000 base pairs (bp); mRNA half-lives of 2, 6, 10, and 14 minutes; and plasmid copy numbers of 10, 100, and 1000 per cell. These were supplemented by deterministic simulations for the same doubling times, at the following parameter values: gene lengths from 10 to 100 in steps of 10 bp, from 100 to 1000 in steps of 100 bp, and from 1000 to 10000 in steps of 1000 bp; mRNA half-lives from 2 to 14 minutes in steps of 1 minute; and plasmid copy numbers from 1 to 9 in steps of 1, from 10 to 100 in steps of 10, and from 100 to 1000 in steps of 100 copies per cell.

Similar to the parameter sweeping carried out for the stochastic simulations, we used the deterministic simulation results for each parameter set to calculate the RNA polymerase-promoter binding on rate, k_on, at which there will be a five percent difference between the models with and without a representation of the host cell genome; for the deterministic results, the 5% threshold was determined using the fzero function in MATLAB, which searches for a zero-crossing between two given points.

### Interpolation of on-rate thresholds

The on-rate (k_on) threshold above which a 5% deviation between the genome and no-genome models occurred has been calculated explicitly only at the set of parameter values listed above (based on stochastic simulations supplemented by cross-validated deterministic simulations to increase the density of the sampling of parameter space). To allow the k_on threshold to be calculated at values other than those explicitly simulated, we created a MATLAB script to carry out the necessary interpolation using a local minimization method. In local linear fitting, to find the unknown point at a desired parameter value, one draws a straight line connecting the known points on either side of the desired value, and takes the point on that straight line as the interpolated result at the desired parameter value. Note that this process minimizes the total distance between the interpolated point and the two known points, and we use this idea to perform our interpolation in our 5-dimensional space (k_on as a function of four parameters: growth rate, gene length, mRNA half life, and plasmid copy number). For any single given 4-dimensional parameter set, the nearest available set of parameter values is determined by finding the two nearest parameter values in each direction on this 4-dimensional mesh; combining all four dimensions yields the 16 nearest points on the mesh. Since these 16 data points do not generally fit well to a linear function, we obtain the interpolated on-rate value for a given parameter set by searching for the k_on value that minimizes the total distance in 5-dimensional space to those nearest 16 points, using the MATLAB fminsearch function to carry out the minimization operation.

**Figure 5:**
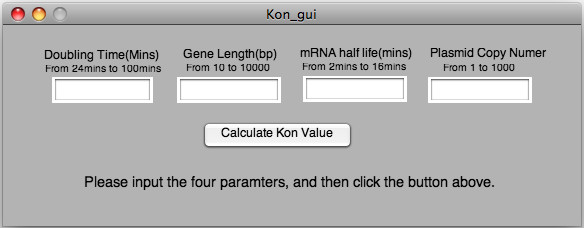
**MATLAB graphical user interface**. MATLAB graphical user interface. The on-rate at which excluding the genome yields less than a 5% difference between the genome and no-genome models is a complex function of the parameters: population doubling time, gene length, mRNA degradation half-life, and plasmid copy number. This space is sampled only at discrete points, but the MATLAB user interface (provided in the additional files accompanying this paper) allows the user to enter any value within the ranges sampled by our simulations (the allowable range is specified above each parameter's input window). A threshold on-rate (above which the genome and no-genome models differ by less than 5%) is calculated by a minimum-distance interpolation between the nearest available points (see text for more detail).

The above interpolation has been implemented in MATLAB script that presents a simple user interface allowing the user to enter the desired parameter values (within the ranges spanned by the simulations), after which the script will carry out the interpolation for the given point and return the k_on value above which a 5% difference arises between the genome and no-genome models: any promoter on-rate constant larger than this predicted value can exclude the computationally expensive genome from the simulations without creating more than a five-percent error, while any constant smaller than this should include the genome. The user interface is shown in Figure 5, and the MATLAB files required to implement it are provided along with this paper (see Additional File 2).

## Results and discussion

### Percent difference of reporter transcript averages between models

**Figure 6:**
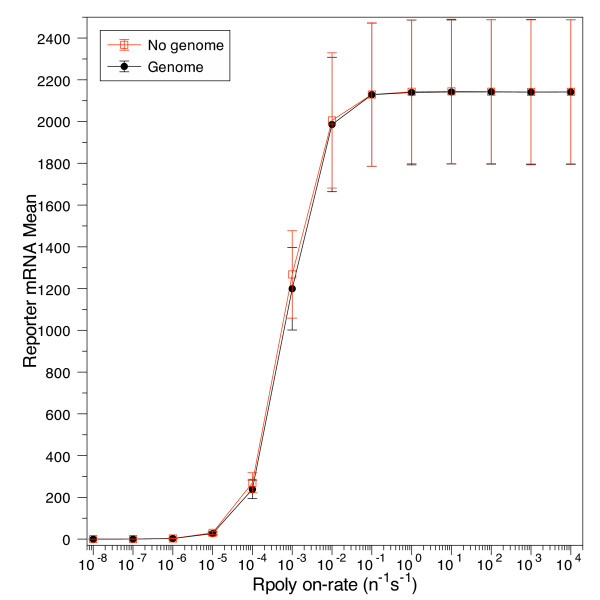
**The effect of the genome on the reporter transcript output**. The effect of the genome on the reporter transcript output. At small enough binding constants neither model is able to produce a significant number of transcripts: the average time between transcriptions is much larger than the doubling time, leading to an average of much less than one transcript per cell division. As the binding constant increases, the reporter promoter starts to compete with the genomic promoters for RNA polymerase, ultimately producing a difference in the number of transcripts between models. The above example has been arbitrarily chosen; it uses the same parameters as in Figure 2 (doubling time = 24 min; plasmid copy number = 10; gene length = 10 bp; and mRNA half-life = 6 min). The error bars are a single standard deviation in the transcript number distributions generated by the stochastic simulations.

As shown in Figure 3, using the stated parameters as a representative example, the percent difference of reporter transcripts between models changes as a function of binding constant between RNA polymerase and the target promoter (k_on). An excessively small binding constant (≈ 10^-10^n^-1^s^-1 ^to 10^-7 ^n^-1^s^-1^) prevents the RNA polymerase from binding to the promoter, thereby producing an insignificant number of transcripts, usually less than one per cell division, as shown in Figure 6. The constant can be so small that noise dominates the system, leading to essentially random results, including some in which more reporter transcripts are produced in simulations that use the genome, relative to the simulations that only use the plasmid-borne reporter genes. Eventually the binding constant becomes large enough to produce a considerable quantity of transcripts; at this point the genome's presence competes with the reporter gene for access to RNA polymerase and reduces the transcription of the reporter gene, producing a significant percent difference between models. As the binding constant to the reporter promoter further increases, the RNA polymerase binding saturates and the promoter generates nearly the same number of transcripts with or without the presence of the competing genome; the difference between models trends towards zero as the binding constant approaches infinity.

Figure 3 shows there are two binding constant ranges for each set of parameters where there is less than a 5% difference in transcript production. We have not considered the lower range, here, because of the insignificant number of transcripts produced, usually an average of much less than one per cell division. In this regime, the two versions of the model are both matching simply because they are both yielding a result of "nearly zero." For the case we wish to consider, that of observing the output of a target gene through the expression of a reporter, such low levels of transcription would be invisible to current detection techniques, requiring single-molecule resolution against the noisy background of the cytoplasm, and thus for the moment we consider it justified to exclude this near-zero range in our simulations. The higher k_on rate constant limit corresponds to transcript numbers on the order of 10^2 ^to 10^4^, a magnitude that is much more amenable to experimental access and thus potentially more significant for use in other studies.

### Accuracy of the interpolated on-rates

To test the accuracy of the interpolated on-rates, the on-rates were entered back into both versions of the model and run for 30 different simulations seeds for a duration of 30 cell divisions, after creating steady state values for all species within the model. The percent differences were assembled and statistically compared to the population mean of 5% using a level of significance α = 0.95. This process was repeated for all 240 different kinetic situations generated using the stochastic simulations. There was no statistical difference between the population mean and the sample mean obtained from the simulations (data not shown), thereby ensuring that the interpolated values are the correct ones for producing a percent difference of 5%.

### Time reduction via genome exclusion

Excluding the genome from simulation studies does reduce CPU simulation time in the computationally intensive fully stochastic simulations. To illustrate this, the verification runs were used for comparison between models; these simulations employ the same kinetic parameters and duration, and offer a large population size (since each run was repeated multiple times with varying random seeds).

**Figure 7:**
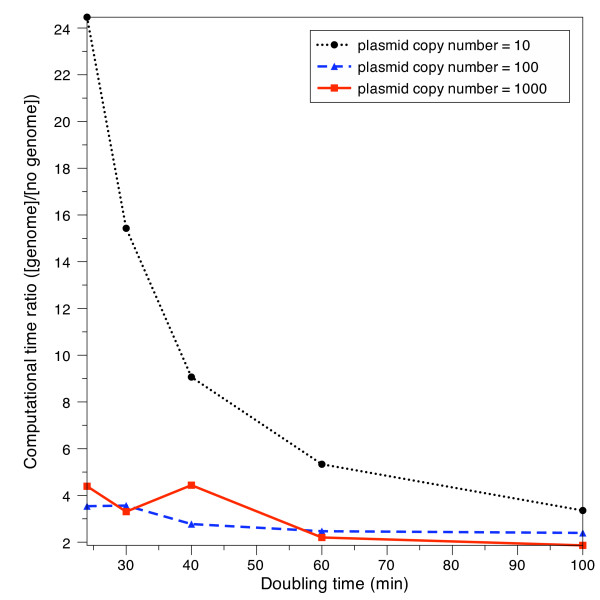
**Ratios of simulation times in models with and without the genome included**. The ratio of simulation time between models with the genome to those excluding it, as a function of doubling time and plasmid copy number. Removing the genome from simulation studies can be 2 to 24 times more efficient compared to those that include it. The data was constructed by averaging the simulation times for all verification runs that employed the set doubling times and plasmid copy numbers, regardless of mRNA half-life and gene length. All computer simulation times were normalized with respect to the computer's CPU strength. The trends suggest that the ratio will approach 1 for sufficiently long doubling times.

Dividing the average run time of the genome by those models excluding it produces a direct measure of the benefit of excluding the host cell genome in the simulations. As Figure 7 shows, computational time can be reduced by a factor ranging from two to 24-fold. Accurate analysis of the time saved between models requires standard CPU power. The verification simulations in this study have been spread out over many computers, most of which have different CPUs. To normalize the results, 10 replicates of a standard run with the same kinetic parameters, duration and random number seed was run (with minimal other processor load) on each type of CPU, for each version of the model. The simulation duration was set to take approximately 30 minutes of CPU time, to average away any aberrations caused by minor fluctuations in CPU availability over time. The run durations for these standard runs were then used to create a scaling factor for each CPU type, and the simulation times reported in Figure 7 were corrected by these factors.

**Figure 8:**
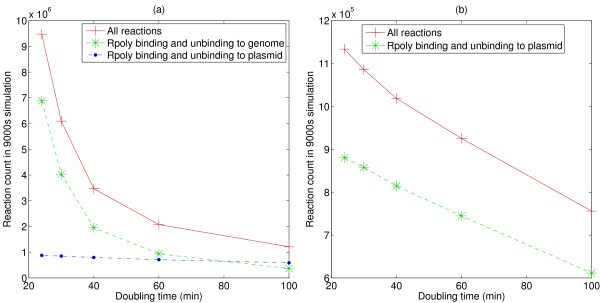
**Number of reaction steps simulated**. Fixing three of the input parameters (plasmid copy number is 10, mRNA half-life is 6 min, and gene length is 1000 bp), we plot the number of reaction steps taken in a stochastic run simulating 9000 seconds of time. (A) With-genome model. The total number of reaction steps, and the number of reactions dedicated to RNA polymerase binding/unbinding to the genomic operons, and to the plasmid carrying our gene of interest. (B) No-genome model. The total number of reaction steps, and the number of reactions dedicated to RNA polymerase binding/unbinding to the plasmid carrying our gene of interest.

The simulation spends most of its time on the RNA polymerase binding/binding reactions: the reactions operon_ns+Rpoly, operon_s+Rpoly, and plas+Rpoly in the with-genome model, and simply plas+Rpoly in the no-genome model. Figures 8A and 8B show the number of reaction steps simulated in the with-genome and no-genome versions of the model (keeping plasmid copy number, mRNA half-life, and gene length fixed, while varying cell doubling time). As Figure 8A shows, the number of reaction steps dedicated to simulating the genomic RNA polymerase binding operations falls off more rapidly with growth rate than does the number of steps required to simulate the plasmid-to-RNA polymerase binding. Figure 8B shows that the number of reaction steps simulated in the no-genome version of the model falls off as a function of growth rate, but less rapidly than in the with-genome case; this is the cause of the reduction in the relative advantage of the no-genome version as the growth rate increases, seen in Figure 7. For large plasmid copy numbers, the RNA polymerase binding steps are more time-consuming in the no-genome version of the model, and the computational advantage of excluding the genome is correspondingly smaller; again, this is seen in Figure 7.

### Relationship between the parameters

**Figure 9:**
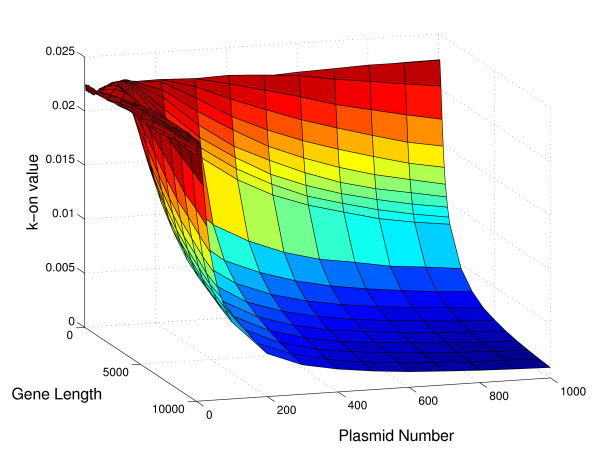
**Dependence of promoter strength on gene length and plasmid number**. The full set of simulations yields promoter strengths, k_on, as a function of four input parameters (gene length, plasmid number, mRNA half-life, and cell doubling time). Here, we fix the doubling time at 30 minutes and the mRNA half-life at 8 minutes, and plot k_on as a function of the two remaining parameters: gene length and plasmid copy number.

**Figure 10:**
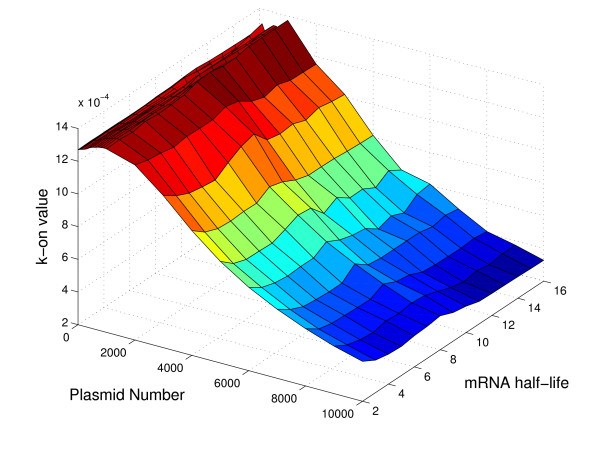
**Dependence of promoter strength on plasmid number and mRNA half-life**. The full set of simulations yields promoter strengths, k_on, as a function of four input parameters (gene length, plasmid number, mRNA half-life, and cell doubling time). Here, we fix the doubling time at 30 minutes and the gene length at 4000 base pairs, and plot k_on as a function of the two remaining parameters: plasmid copy number and mRNA half-life.

**Figure 11:**
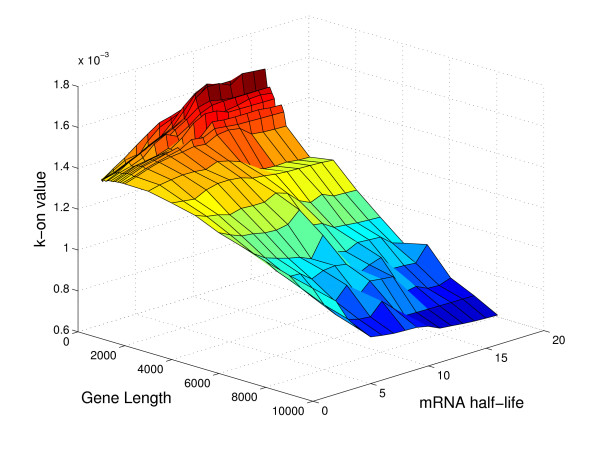
**Dependence of promoter strength on mRNA half-life and gene length**. The full set of simulations yields promoter strengths, k_on, as a function of four input parameters (gene length, plasmid number, mRNA half-life, and cell doubling time). Here, we fix the doubling time at 30 minutes and the plasmid copy number at 200, and plot k_on as a function of the two remaining parameters: mRNA half-life and gene length.

Figures 9, 10, and 11 show the dependence of the k-on value on gene length, plasmid number and mRNA half-life, while the doubling time is fixed at 30 minutes. These plots are 3D slices through the full 5D space of results (where the five dimensions are the four input parameters, mRNA half life, gene length, plasmid number, and doubling time, and the output promoter on-rate, k_on). The plots show some of the nonlinearity inherent in the relationship of k_on to the parameters, and help to indicate why it has not proven to be possible to reduce the parameter relationships to a single regression equation.

### Potential extensions

Simulating the translation of mRNA to protein, downstream of the transcriptional events discussed here, requires a significantly more elaborate model [10] with correspondingly greater computational demands. One extension of this study would be to investigate the binding on-rates for ribosomes binding to the ribosome-binding-sites (RBS) of the mRNA binding sites, and once again compare the results when the presence of the genome is modelled to those when it is excluded; presumably there would be a similar possibility of excluding the representation of the genome under some parameter ranges (where the main parameters would remain the same: doubling time, gene length, mRNA half-life, and plasmid copy number). Since translation follows transcription in the gene expression process, the range of parameter values in which one can exclude the genome from studies of the translational output of a target gene should be smaller than the regions found in the current study of transcriptional output: the system will be subject to the constraints imposed by matching the transcriptional results, as well as additional constraints required to match the translational results.

The ability of RNA polymerase to produce an approximately equal amount of transcripts at large enough binding constants for both models raises an important question: are there enough RNA polymerases left when a large rate law exists for the reporter promoter to transcribe the necessary genomic genes for cell division? The presence of a large rate for the reporter transcript will produce metabolic strain on the cell [54-56], possibly leading to an increase in doubling time that is not captured within the current model. Further studies on modelling the effect of metabolic strain and its feedback with cellular doubling time will help to clarify this issue.

## Conclusion

Efforts to create accurate, quantitative models of *Escherichia coli *genomic networks using chemical equations results in large reaction schemes, with reactions potentially proceeding at a wide range of rates. The large computational time required to simulate these reactions is a persistent problem for large-scale cellular simulation. To help address one aspect of this problem, we have investigated the necessity of simulating the presence of the *E. coli *genome when studying a target gene inserted on a plasmid. The presence of the genome, introduced using our "mean-field" approach, is felt by the target gene through the competition for free RNA polymerases available to bind to the target gene's promoter and generate transcripts. However, there are ranges of the parameter space in which the presence of the genome yields a negligible difference in the number of reporter transcripts produced from the target gene, and in these cases is it possible to exclude any explicit representation of the genome and save the computations required to simulate the associated additional reactions. Stochastic simulations show speed increases of from two to 24 times, when the genome is excluded from our models. We have generated a set of fully stochastic simulations and found the promoter on-rate values for which the genome and no-genome models differ by less than 5%, and augmented these stochastic simulations with cross-validated deterministic runs to increase the number of sampled points in parameter space. Within the ranges of our four independent parameters (growth rate, gene length, mRNA degradation half-life, and plasmid copy number), we have produced a MATLAB user interface that will allow the user to input any set of parameters and obtain the promoter on-rate value (k_on) above which the effect of the genome will fall below our 5%-difference threshold. Given the increasing computational demands of cellular simulations, we hope that this approach will aid in the efficiency of other studies, and suggest other methods in which portions of the full cellular system may be excluded without significantly affecting the final results.

## Authors' contributions

MAJI conceived of the study, designed molecular simulations, implemented stochastic simulations, compiled data, analyzed results and drafted the manuscript. GQD carried out deterministic simulations, compiled data, analyzed results, implemented networked runs of stochastic simulations, and produced the MATLAB parameter interpolation routines and user interface. DRM participated in design of the study, and helped draft and finalize the manuscript and revisions. All authors participated in the writing and approved of the final form of the manuscript.

## Appendix

Below is a detailed explanation of the gene expression model, expanding on the information presented in the Methods section. A full list of kinetic parameters for each reaction is provided in Iafolla and McMillen [10].

### Nomenclature

The following is a complete list of species names used in the model:

**Table 4:** Species used in both versions of the model

*Species Name*	*Species*
Rpoly	RNA polymerase
Rpoly_reporter	RNA polymerase elongating the reporter mRNA transcript from the reporter gene
closed_Rpoly_prom_reporter	RNA polymerase in a closed-complex with the repoter promoter
deg_mRNA_incom_reporter	Nascent reporter mRNA degradation product
deg_mRNA_reporter	Reporter mRNA degradation product
incom_mRNA	Nascent reporter mRNA
mRNA_reporter	Reporter mRNA
open_Rpoly_prom_reporter	RNA polymerase in an open-complex with an mRNA reporter promoter
plas	Promoter on the plasmid
v	Counter (representing cell volume)
**Species used exclusively in the model containing the genome**	
Rpoly_operon_ns	RNA polymerase elongating an average mRNA transcript from a template operon
Rpoly_operon_s	RNA polymerase elongating an average RNA transcript from a template operon
closed_Rpoly_prom_ns	RNA polymerase in a closed-complex with an mRNA operon promoter
closed_Rpoly_prom_s	RNA polymerase in a closed-complex with an mRNA operon promoter
mRNA	Average mRNA (gene length)
mRNA_small	Approximately 90% of the average mRNA; all species names that include "small" refer to this shorter species and its products/complexes
open_Rpoly_prom_ns	RNA polymerase in an open-complex with an mRNA operon promoter
open_Rpoly_prom_s	RNA polymerase in an open-complex with a stable RNA operon promoter
operon_ns	Average mRNA operon
operon_s	Average stable RNA operon
stable_RNA	Average stable RNA (full operon length)

The cellular processes represented in the model are discussed individually, below:

#### Cellular division

To reflect the exponential growth of bacterial cells in a nutrient-rich liquid culture, we include cell growth and division, incorporated as a process that grows to a threshold volume and is then halved. At division, all species have their numbers cut approximately in half: for large numbers, a binomial distribution is used to calculate the new number, while small numbers (less than 100) have each molecule explicitly checked and randomly assigned to a daughter cell with equal probability [26]. The model follows only one cell as a representative of the full population, so the second daughter effectively vanishes after division. Tracking such a representative cell over long times yields the same statistics as tracking an ensemble of many cells over shorter times, if we make the reasonable assumption that the system is ergodic.

Cell volume is represented by a "counter" species, *v*, whose exponential growth is governed by the following reaction, with rate constants adjusted to produce various doubling times to match the experimental conditions being examined:

(R1) *v *→ 2*v *

For a doubling time τ, the rate constant is set to k = ln(2)/τ. The reaction is initialized at *v *= V_0_, and cellular division occurs when *v *reaches 2V_0_. Our model treats all processes as stochastic, but the resulting degree of variability depends strongly on the number of molecules participating in the reaction. The range of cell division times can thus be tuned by the choice of V_0_; here we set V_0 _= 1000, which yields a very slight degree of variability in the cell division times. This variability arises from two sources: the stochastic rate of reaction R1, and the random assortment of the counter *v *between daughter cells at division: *v *is cut only approximately in half at cell division, like all other species, and thus the initial volume after cell division lies in a small range around V_0_.

#### Enzyme binding, unbinding, isomerization and clearance

Since the only enzymes used in this model are RNA polymerases only binding to promoters need consideration. The bimolecular reactions for RNA polymerase (Rpoly) binding to a promoter on a gene (plas or operon) are shown below:

(R2) Rpoly + plas ⇔ closed_Rpoly_prom   K_R2 _= [k_on_Rpoly/(*v*/V_0_)]/k_off_Rpoly

(R3) closed_Rpoly_prom → open_Rpoly_prom   k_R3 _

RNA polymerase initially forms a closed-complex with the promoter region, which then undergoes isomerization (R3) into an open-complex. The rate constants for R2 and R3 are adjacent to the reactions; that for R2 is scaled to mimic dilution of cell cycle progression: as the cell grows, the increase in volume decreases the probability of the two species coming into contact and reacting, effecting reducing the rate constant [26]; this effect is incorporated by dividing the rate constants by *v*/V_0_.

Following binding, the enzyme clears the promoter at a particular rate. The elementary reactions for this process are shown below:

(R4) open_Rpoly_prom → Rpoly_operon + operon + incom_mRNA

We create a nascent transcript (mRNA_incom) at this step to allow subsequent translation to proceed; this feature will prove very helpful in studying future simulated studies of protein synthesis. Reaction R4 also shows an important assumption: the regeneration of a binding site after clearance allows another enzyme to bind to the same gene, creating the multiple simultaneous elongation processes observed in actual bacterial cells.

#### Elongation

To avoid the computational complexity of accounting for all elongating intermediates (growing mRNA and peptides of every possible length), the following approximation has been employed: a single intermediate is converted to the final product at a rate corresponding to the average time taken by the complete polymerization process. Using average elongation rates for specific cell growth rates as specified by Bremer and Dennis [12], the elongating species produce only the enzyme and the polymerized product, not the template that is read. This is shown below in Reaction R5:

(R5) Rpoly_operon → Rpoly + mRNA

Compliment to this reaction is the disappearance of the nascent transcript made available during transcription: incom_mRNA→(), where () is a null placeholder. The elongation rate constant can be summarized as k_elongation _= ρ/λ, where ρ and λ are the polymerization rate and length of template, respectively.

#### Enzyme and genome production

Many processes involved in molecular biology are either too complex to model or not characterized at present. In our model, we use simplified zeroth-order production rates for complicated species involved: although the assembly details of some species are not fully available, there is considerable information on population size of these species. In *E. coli*, the average number of RNA polymerases and genome equivalents per cell are known at several cellular growth rates [12], and their production is represented by the elementary reactions below:

(R6) () → Rpoly

(R7) () → plas

(R8) () → operon

The operon species in R8 is representative of the genome, since our model employs RNA polymerase binding directly to the promoter sequence of the average operon. The rate constant for production can be summarized as k_rep _= (ν/1.5)/τ, where ν and τ are the average number per cell and cellular doubling time, respectively.

#### mRNA degradation

The presence of RNases in *E. coli *implies that mRNA possess a finite life-span. The following reactions are used to represent mRNA degradation:

(R9) mRNA_reporter → ()

(R10) incom_mRNA_reporter → ()

For a half-life *h*, the rate constant for R9 and R10 is set to k = ln(2)/*h*.

We assume that RNases can degrade nascent transcripts. To account for degrading a transcript while it is being created we propose the following elementary reaction and rate constant:

(R11) Rpoly_mRNA_reporter → Rpoly

*k*_R11 _= incom_mRNA_reporter·*k*_mRNA_degradation_/Rpoly_mRNA_reporter

The reaction indicates that an RNA polymerase currently producing a transcript becomes an unscathed RNA polymerase and a degraded mRNA. Although this reaction implies that all RNA polymerases producing a transcript are subject to degradation, the proportionality to incomplete transcripts is specified in the rate constant. The Rpoly_mRNA species present in the denominator of the rate constant makes the reaction rate independent of the number of elongating RNA polymerases.

#### Modelling RNA production from operons

We assume that all genes in our relevant genome are clustered into operons. Our model creates a single transcript for the entire operon, mimicking the *lac *operon [57]. To make the elementary reactions simple and accurate for mRNA and subsequent peptide production, RNA polymerase binds once to the promoter and produces a transcript of average length under corresponding kinetics; the ejection of the mRNA occurs simultaneously with RNA polymerase transcribing the adjacent gene on the operon, or in the case of the last gene on the operon, being released. This is shown in the following reactions for a hypothetical three gene operon, where the binding (R2), isomerization (R3) and clearance steps (R4) have been omitted:

Rpoly_operon1 → Rpoly_operon2 + mRNA   k = k_transcription_

Rpoly_operon2 → Rpoly_operon3 + mRNA   k = k_transcription_

Rpoly_operon3 → Rpoly + mRNA   k = k_transcription_

The numeric suffix on the Rpoly_operon species represents the gene number adjacent to the promoter. Notice that the rate constants for the above reactions are all equivalent. The release of the mRNA while the RNA polymerase is still elongating the operon allows ribosomes to bind and perform translation without requiring additional species; the act of transcription is conserved since RNA polymerase only binds once to the promoter. Evidently, the total time to transcribe all three genes is equivalent to the time for transcribing the whole operon.

Contrast to mRNA production, stable RNA is easily produced. Since this RNA is not translated there is no need to include ribosomes translating complete transcripts before the operon is finished elongation. Hence, the length of stable RNA in the model is equivalent to the average stable RNA operon length.

## Supplementary Material

Additional file 1 Bionets files for the models. Files used to generate the stochastic simulations, using the Bionets stochastic simulation tool (required to read the files, and freely available from ). The ZIP file extracts to a directory containing files corresponding to the with-genome and no-genome versions of the model. Click here for file

Additional file 2 MATLAB user interface. Files used to create the MATLAB user interface, allowing the user to enter four parameters (plasmid copy number, gene length, mRNA half-life, and bacterial cell doubling time), and get back the k_on rate above which excluding the genome will make less than a five percent difference in the simulated transcription levels of the plasmid-borne gene of interest. The ZIP file extracts to a directory containing three files that should be placed in the directory where the user interface will be used; the interface may be executed by opening MATLAB and running the script kon_gui.m. Click here for file
